# Presence of *Rickettsia felis* in the Cat Flea from Southwestern Europe^1^

**DOI:** 10.3201/eid0801.010193

**Published:** 2002-01

**Authors:** Francisco J. Márquez, Miguel A. Muniain, Jesús M. Pérez, Jerónimo Pachón

**Affiliations:** * Universidad de Jaén, Jaén, Spain; † Hospital Universitario Virgen Macarena, Seville, Spain; ‡ Hospital Universitario Virgen del Rocío, Seville, Spain

**Keywords:** *Rickettsia*, cat flea, *Ctenocephalides*, Spain, PCR, sequencing, 16S rRNA, ompa, ompb, citrate synthase

## Abstract

*Rickettsia felis*, formerly called ELB agent, was identified by using molecular biology techniques in the cat flea (*Ctenocephalides felis felis*) from southwestern Spain. For the first time this flea-transmitted rickettsia has been detected within its vector in Eurasia.

Members of the genus *Rickettsia* are commonly associated with hematophagous arthropods such as ticks, fleas, or lice. *Rickettsia felis*, formerly ELB agent, was detected in 1990 when tissues from the cat flea, *Ctenocephalides felis,* were examined under electron microscopy. After this, several antigenic and molecular studies concerning this rickettsia were developed (*1*). *R. felis* is maintained in cat fleas by transovarian transmission (*2*). Infection in humans has been described in the USA (*3*), Mexico (*4*), and Brazil (*1*) by polymerase chain reaction (PCR) amplification and recently in France by serologic tests (*1*).

During a study concerning rickettsial organisms transmitted by ticks in southwest Spain, using molecular tools for diagnosis, a rickettsial microorganism was detected in some cat fleas on domestic cats and dogs from different counties of the Cadiz Province.

## The Study

The fleas used in this study (60 females and 11 males) were collected, together with ticks, from 2 cats and 12 dogs from eight localities of Cadiz Province in southwestern Spain from May to August of 1999 (Figure, Table). The hosts were domestic and peridomesticated dogs and cats living in a range of health-care conditions. Collected fleas were fixed in 70% ethyl alcohol and stored at 4°C until they were processed. Taxonomic determination was made by using current taxonomic keys (*5*,*6*). All specimens subjected to analysis were *C. felis* *felis* (Bouché, 1835).

**Figure:**
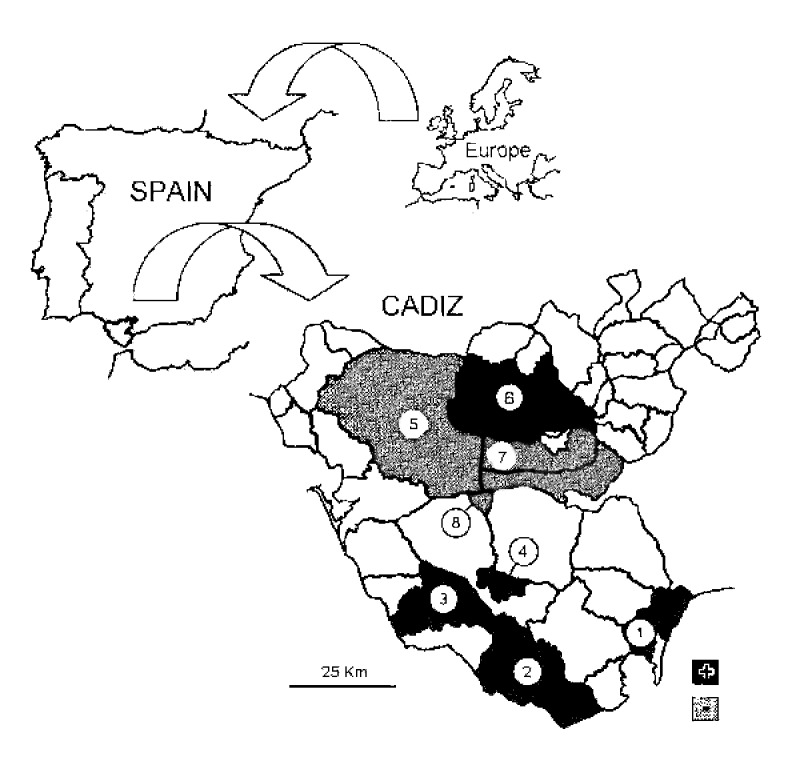
Localization of randomly selected sampling points in Cadiz Province for cat fleas with indication of the presence of *Rickettsia felis* by polymerase chain reaction procedures. Localities are 1. San Roque, 2. Tarifa, 3. Vejer de la Frontera, 4. Benalup, 5. Jerez, 6. Arcos de la Frontera, 7. San José del Valle, 8. Paterna de Rivera.

**Table:** Origin, host range, lot composition, and polymerase chain reaction results of cat fleas (*Ctenocephalides felis felis)*, captured in Cadiz Province, southwestern Spain, used in this study

Locality	Capture date	Host	*C. f. felis* females	*C.f. felis* males	PCR result
Arcos de la Frontera	23/05/1999	dog	2	1	+
Benalup	09/08/1999	dog	7	4	+
Jerez	24/05/1999	dog	2	-	-
Paterna de Rivera	24/05/1999	dog	5	1	-
San José del Valle	24/05/1999	dog	6	-	-
San Roque	21/05/1999	dog	8	-	+
San Roque	21/05/1999	dog	1	-	+
Tarifa	11/06/1999	dog	5	3	+
Tarifa	11/06/1999	dog	1	-	+
Vejer de la Frontera	12/06/1999	dog	3	2	-
Vejer de la Frontera	09/08/1999	cat	7	-	+
Vejer de la Frontera	09/08/1999	cat	4	-	-
Vejer de la Frontera	09/08/1999	dog	5	-	-
Vejer de la Frontera	09/08/1999	dog	4	-	-

DNA was extracted from 14 lots of fleas (ranging from 1 to 11 specimens per lot) by using the DNeasy_Tissue kit(Qiagen GmbH, Hilden, Germany) (*7*). Elution of DNA was made in 100 μL of TE buffer (1 mM Tris HCl, 0.1 mM EDTA). Extraction blanks, consisting of water processed along with flea samples, were also included as controls.

A Biometra DNA Thermalcycler (Gottingen, Germany) was used for all PCR amplification. Three microliters of each DNA extraction were added to 27 μL of master mixture for each reaction. Final reagent concentration was 0.2 μM for each primer, 200 μM for each deoxynucleotide triphosphate (Promega Corp., Madison, WI), 2 U of Biotaq polymerase (BioLine, London, UK), and 1x Bioline buffer. The following thermal cycler parameters were used with the primer pairs for citrate synthase (*glta*) RpCS.877p and RpCS1258n (*8*), 120-kDa genus common antigen (*ompB*) (rfompbf: 5’–GAC AAT TAA TAT CGG TGA CGG, and rfompbr: 5’-TGC ATC AGC ATT ACC GCT TGC), 190-kDa protein antigen (*ompA*) Rr190.70p, and Rr190.602n (*8*): 96°C (90 sec), followed by 35 cycles of 94°C (30 sec), 50°C (30 sec), and 72°C (45 sec), followed by an extension period (72°C, 7 min). For the amplification of a 426-base pair fragment of 16S rRNA gene, we used the primers fD1 (*9*) and Rc16S.452n (*10*) and 59°C as annealing temperature.

Seven lots from five localities around Cadiz and Gibraltar bays were positive to amplification of fragments of 16S rRNA, *glta*, *ompA* and *ompB* genes*.*

In brief, after amplification, primers and nucleotides were removed from 300 μL of PCR products by purification on the Wizard PCR preps purification system (Promega, Madison, WI). Approximately 100 fmol of the purified PCR product (4-5 μL) were used directly in the sequencing reaction.

The PCR cycle sequencing was performed for each amplicon by using the correct forward or reverse primers and the Silver sequence DNA Sequencing System (Promega). Sequencing reaction products were loaded twice on 40 cm 6% polyacrylamide 7M urea gels by electrophoresis in the Sequi-Gen Nucleic Acid Sequencing System (BioRad, Hercules, CA) at 55 W of constant electrophoresis (55°C) and separated for 4 hr 30 min. and 2 hr 30 min, respectively. Gel was silver stained by using the standard Promega protocol. A permanent record was made in scanning the gel. To determine the sequence of positions near primers, we used a 10% polyacrylamide 7M urea electrophoresis gel. The sequence of both strings was determined twice for each fragment.

Sequences obtained were compared with those from other *Rickettsia* species in GenBank by using the BLAST utility (National Center for Biotechnology Information, Bethesda, MD) and FASTA routine from GCG environment. Fragment sequence for 16S rRNA, *glta*, *ompA*, and *ompB* sequence were identical to previously reported sequence for *R. felis.* The 16S rRNA amplified fragment was identical to previously reported sequence (GenBank L28944) between positions 1 and 410 (*3*). The fragment sequenced for citrate synthase corresponded to positions 757 and 1138 in GenBank accession AF210692 (*1*). The fragment amplified for *ompA* corresponded to positions 478 to 987 in GenBank accession AF191026 (*11*). The fragment amplified for *ompB* corresponded to positions 599 to 1259 in GenBank accession AF210695 (*1*). Amplification was unsuccessful in all negative controls.

## Conclusions

*R. felis* has been found extensively in commercial colonies and natural cat fleas, parasitizing a large range of mammalian hosts in several states of the United States (*12*,*13*).

For the first time *R. felis* was detected in Eurasia, by means of PCR and partial sequencing of genes classically used in rickettsial molecular characterization and phylogeny. The sequences of *glta*, *ompA,* *ompB,* and 16S rRNA from Cadiz cat fleas were identical to the homologous sequences previously reported for *R. felis* obtained from fleas reared in EL Laboratories (Soquel, CA) (*3*) and Louisiana State University (*11*) and isolated by Flea Data Inc. (Freeville, NY) (*1*).

In humans, *R. felis* may produce a clinical syndrome similar to murine typhus (*3*). Thus, *R. felis* could be implicated in murine typhus-compatible cases detected in southwest Spain (*14*), especially since the oriental rat flea, *Xenopsylla cheopis* (Rothschild, 1903), is absent from this area.

Thirteen species of flea belonging to the genus *Ctenocephalides* have been described to date (*15*), mainly distributed in continental Africa (*16*), with a worldwide contemporary distribution in a large range of hosts, mainly anthropic species of the group (*C. felis*), which has a large potential host range. The primary source of the bacterium might be Africa, where this flea genus apparently originated.

This work was supported in part by grants from the Fondo de Investigación Sanitaria (FIS-99/0296), Servicio Andaluz de Salud (SAS-203/98) of Junta de Andalucía, and Universidad de Jaén (UJA-FLV/jbf) research programs.
